# Quantitative Evaluation of ^18^F-Flutemetamol PET in Patients With Cognitive Impairment and Suspected Alzheimer's Disease: A Multicenter Study

**DOI:** 10.3389/fneur.2020.578753

**Published:** 2021-01-13

**Authors:** Hiroshi Matsuda, Kengo Ito, Kazunari Ishii, Eku Shimosegawa, Hidehiko Okazawa, Masahiro Mishina, Sunao Mizumura, Kenji Ishii, Kyoji Okita, Yoko Shigemoto, Takashi Kato, Akinori Takenaka, Hayato Kaida, Kohei Hanaoka, Keiko Matsunaga, Jun Hatazawa, Masamichi Ikawa, Tetsuya Tsujikawa, Miyako Morooka, Kenji Ishibashi, Masashi Kameyama, Tensho Yamao, Kenta Miwa, Masayo Ogawa, Noriko Sato

**Affiliations:** ^1^Integrative Brain Imaging Center, National Center of Neurology and Psychiatry, Kodaira, Japan; ^2^Department of Radiology, National Center of Neurology and Psychiatry, Kodaira, Japan; ^3^Cyclotron and Drug Discovery Research Center, Southern TOHOKU Research Institute for Neuroscience, Koriyama, Japan; ^4^Innovation Center for Clinical Research, National Center for Geriatrics and Gerontology, Obu, Japan; ^5^Department of Radiology, Kindai University Faculty of Medicine, Osakasayama, Japan; ^6^Division of Positron Emission Tomography, Institute of Advanced Clinical Medicine, Kindai University Hospital, Osakasayama, Japan; ^7^Department of Molecular Imaging in Medicine, Osaka University Graduate School of Medicine, Suita, Japan; ^8^Biomedical Imaging Research Center, University of Fukui, Fukui, Japan; ^9^Department of Neuro-Pathophysiological Imaging, Graduate School of Medicine, Nippon Medical School, Kawasaki, Japan; ^10^Department of Radiology, Medical Centre Omori, Toho University, Tokyo, Japan; ^11^Team for Neuroimaging Research, Tokyo Metropolitan Institute of Gerontology, Tokyo, Japan; ^12^Department of Radiology, National Center for Geriatrics and Gerontology, Obu, Japan; ^13^Joint Research Division for the Quantum Cancer Therapy, Research Center for Nuclear Physics, Osaka University, Osaka, Japan; ^14^Department of Neurology, Faculty of Medical Sciences, Fukui, Japan; ^15^Department of Radiology, Tokyo Metropolitan Geriatric Hospital, Tokyo, Japan; ^16^Preparing Section for New Faculty of Medical Science, Fukushima Medical University, Fukushima, Japan

**Keywords:** amyloid imaging, positron emission tomography, Alzheimer's disease, ^18^F-flutemetamol, standardized uptake value ratio, centiloid scale

## Abstract

**Background:** In clinical practice, equivocal findings are inevitable in visual interpretation of whether amyloid positron emission tomography (PET) is positive or negative. It is therefore necessary to establish a more objective quantitative evaluation method for determining the indication for disease-modifying drugs currently under development.

**Aims:** We aimed to determine cutoffs for positivity in quantitative analysis of ^18^F-flutemetamol PET in patients with cognitive impairment and suspected Alzheimer's disease (AD). We also evaluated the clinical efficacy of amyloid PET in the diagnosis of AD. This study was registered in the Japan Registry of Clinical Trials (jRCTs, 031180321).

**Methods:** Ninety-three patients suspected of having AD underwent ^18^F-flutemetamol PET in seven institutions. A PET image for each patient was visually assessed and dichotomously rated as either amyloid-positive or amyloid-negative by two board-certified nuclear medicine physicians. If the two readers obtained different interpretations, the visual rating was rerun until they reached consensus. The PET images were quantitatively analyzed using the standardized uptake value ratio (SUVR) and standardized Centiloid (CL) scale with the whole cerebellum as a reference area.

**Results:** Visual interpretation obtained 61 positive and 32 negative PET scans. Receiver operating characteristic analysis determined the best agreement of quantitative assessments and visual interpretation of PET scans to have an area under curve of 0.982 at an SUVR of 1.13 and a CL of 16. Using these cutoff values, there was high agreement between the two approaches (kappa = 0.88). Five discordant cases had SUVR and CL values ranging from 1.00 to 1.22 and from 1 to 26, respectively. In these discordant cases, either diffuse or mildly focal elevation of cortical activity confused visual interpretation. The amyloid PET outcome significantly altered the diagnosis of AD (χ^2^ = 51.3, *p* < 0.0001). PET imaging elevated the proportions of the very high likelihood category from 20.4 to 46.2% and the very low likelihood category from 0 to 22.6%.

**Conclusion:** Quantitative analysis of amyloid PET using ^18^F-flutemetamol can objectively evaluate amyloid positivity using the determined cutoffs for SUVR and CL. Moreover, amyloid PET may have added value over the standard diagnostic workup in dementia patients with cognitive impairment and suspected AD.

## Introduction

One of the pathological features of Alzheimer's disease (AD) is senile plaques caused by the aggregation and accumulation of amyloid beta (Aβ) in the brain. Positron emission tomography (PET)-mediated imaging of Aβ in the brain (amyloid PET) is expected to be useful for improving accuracy in the differential diagnosis of AD and non-AD conditions and for developing disease-modifying drugs. In Japan, one of the approved tracers for amyloid PET imaging is ^18^F-flutemetamol (1, 2). This radiopharmaceutical, delivered as a final product to a clinical facility, has proven efficacy in the visualization of Aβ plaques in the brains of patients with cognitive impairment and suspected AD (https://www.nmp.co.jp/sites/default/files/member/vizamyl/pdf/reference.pdf).

When using amyloid PET in clinical practice, qualitative determination of whether amyloid PET is positive or negative is performed by visual interpretation alone. In this binary classification, equivocal findings are inevitable and lead to interrater variability in visual interpretation (3, 4) because each rater has his or her own experience and potential internal criteria. Equivocal findings should be avoided for determining the indication for disease-modifying drugs currently under development. Accordingly, quantitative analysis has been proposed as an adjunct to visual interpretation (5–9). In the quantitative analysis of amyloid PET, standardized uptake value ratio (SUVR) has been widely used. However, SUVR values vary not only according to the target and reference regions used but also according to the particular amyloid tracer used. This variation could be resolved through a Centiloid (CL) scaling process that standardizes the quantitative amyloid imaging measures by standardizing the outcome of each analysis method or PET ligand to a scale from 0 to 100 (10). The CL scaling method may facilitate direct comparison of results across institutions even when different analysis methods or tracers are used and may enable cutoffs for amyloid positivity to be clearly defined. Although the CL scaling method has been applied to ^18^F-flutemetamol PET (11), this method has not been compared with visual assessments, and its cutoff for amyloid positivity has not been determined.

The aim of this multicenter study was to determine the cutoffs for amyloid positivity for SUVR and CL in quantitative analysis by investigating the agreement between quantitative and visual assessments (positive or negative) of ^18^F-flutemetamol PET in patients with cognitive impairment and suspected AD. We also aimed to evaluate the clinical efficacy of amyloid PET in the diagnosis of AD.

## Materials and Methods

### Participants

In total, 103 Japanese patients (46 men and 57 women; age range, 36–91 years) were recruited from seven participating centers with a specialized section for dementia (Table 1). General cognition was assessed using the Mini-Mental State Examination (MMSE) (12). Dementia severity was scored using the global Clinical Dementia Rating (CDR) (13).

**Table 1:** Details of the PET imaging and image reconstruction methods in each center.

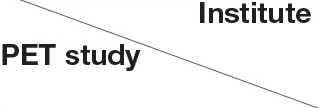		**Kodaira**	**Obu**	**Osakasayama**	**Suita**	**Eiheiji**	**Kawasaki, Tokyo (Ota)**	**Tokyo (Itabashi)**
**Number of patients studied**		**24**	**25**	**15**	**10**	**10**	**5**	**4**
PET imaging	Scanner	Siemens Biograph 16 Truepoint	Siemens Biograph 16 Truepoint	GE Discovery PET/CT 710	Shimadzu Eminence SOPHIA SET-3000 BCT/X	GE Signa PET/MR	Siemens Biograph mCT Flow	GE Discovery PET/CT 710
	Detector	Lu_2_SiO_5_	Lu_2_SiO_5_	Lutetium-based scintillator	Bismuth germanate	Silicon phtomultiplier (lutetium-based scintillator)	Lu_2_SiO_5_	Lutetium-based scintillator
	Attenuation correction	CT	CT	CT	^137^Cs	MRI (zero-TE)	CT	CT
	Injection dose	215 ± 33 MBq	265 ± 43 MBq	290 ± 27 MBq	310 ± 50 MBq	310 ± 25 MBq	218 ± 14 MBq	263 ± 50 MBq
	Start time	61.2 ± 0.8 min	60.4 ± 1.4 min	63.2 ± 3.6 min	60.1 ± 0.3 min	60.0 ± 0.0 min	60.2 ± 0.4 min	60.0 ± 0.0 min
	Scan time	20 min	20 min	20 min	20 min	20 min	20 min	20 min
	Acquistion mode	List mode	List mode	List mode	List mode	List mode	List mode	List mode
Image reconstruciton	Correction for random coincidence counting	Model-based method	Model-based method	Model-based method	Deconvolution method	Model-based method	Model-based method	Model-based method
	Image reconstruction	3D-OSEM	2D-OSEM	3D-OSEM + time-of-flight	2D-OSEM	3D-OSEM (VUE-point) + time-of-flight	3D-OSEM + time-of-flight	3D-OSEM + time-of-flight
	Iteration/subset	4/21	4/16	5/16	4/16	3/16	4/16	4/16
	Z axis filter	–	–	Standard	–	Standard or Light	Standard	Standard
	Post filter	All-pass 4-mm FWHM	All-pass 4-mm FWHM	Gaussian 4-mm FWHM	Gaussian 5-mm FWHM	Gaussian 4-mm FWHM	Gaussian 4-mm FWHM	Gaussian 4-mm FWHM
	Voxel size	2.06 mm (X) × 2.06 mm (Y) × 2.03 mm (Z)	2.04 mm (X) × 2.04 mm (Y) × 2.0 mm (Z)	2.0 mm (X) × 2.0 mm (Y) × 3.27 mm (Z)	2.0 mm (X) × 2.0 mm (Y) × 3.25 mm (Z)	2.0 mm (X) × 2.0 mm (Y) × 2.78 mm (Z)	0.47 mm (X) × 0.47 mm (Y) × 7.0 mm (Z)	2.0 mm (X) × 2.0 mm (Y) × 3.27 mm (Z)
	Matrix size	168 pixels × 168 pixels × 81 slices	168 pixels × 168 pixels × 109 slices	128 pixels × 128 pixels × 47 slices	128 pixels × 128 pixels × 79 slices	128 pixels × 128 pixels × 89 slices	400 pixels × 400 pixels × 21 slices	128 pixels × 128 pixels × 47 slices

Inclusion criteria were as follows: diagnosis of probable or possible AD according to the National Institute on Aging and the Alzheimer's Association (NIA-AA) (14) or diagnosis of major or mild neurocognitive disorder according to the Diagnostic and Statistical Manual of Mental Disorders—V (15) and having undergone brain magnetic resonance imaging (MRI; T1-weighted, T2-weighted, and FLAIR imaging) up to 60 days before registration. The diagnosis was made without the use of amyloid PET imaging.

Exclusion criteria were as follows: no cognitive decline; gross lesions, such as a brain tumor, cerebrovascular malformation, or cortical infarction on MRI; severe allergy to alcohol or polysorbate 80 (solvent); and pregnancy or suspected pregnancy and currently breastfeeding.

Five patients were excluded during screening based on the inclusion or exclusion criteria (no MRI data, four patients; gross lesion on MRI, one patient). Three patients who passed the screening withdrew their consent before the PET scan because of their physical condition. One patient who passed the screening withdrew consent immediately after the PET scan began due to intolerance. Ninety-four patients (43 men and 51 women; age range, 36–91 years) completed the amyloid PET studies. After reevaluation of global CDR, one patient with CDR 0 despite subdomain scores of 0.5 was removed from further analysis. Finally, 93 patients (42 men and 51 women; age range, 36–91 years) were included in this study.

### PET Imaging

One of the seven participating institutions requested that the PET study be performed by another facility. ^18^F-flutemetamol was injected intravenously as a slow bolus in an antecubital vein at a mean ± SD dose of 270 ± 51 MBq (range, 182–370 MBq). A 20-min list-mode PET scan was started from 61 ± 2 min (range, 60–73 min) according to the imaging acquisition guidelines of the Vizamyl® package insert (https://www.accessdata.fda.gov/drugsatfda_docs/label/2017/203137s008lbl.pdf), which recommends a PET scan start time of 60–120 min after Vizamyl® injection. In all participating institutions, all appropriate corrections, including scatter and time-of-flight, were applied with a low-dose computed tomography scan or MRI for attenuation correction (Table 1). Images were reconstructed using the ordered subsets expectation maximization (OSEM) method. Clinical status was checked before and after PET scanning in each participant. Patients were observed for adverse events from the administration of tracer and immediately after the PET scan.

### Visual Interpretation and Quantitative Image Analysis

A static 20-min PET image for each patient was visually assessed and dichotomously rated as either amyloid-positive or amyloid-negative by two board-certified nuclear medicine physicians (HM and YS). Both had completed the electronic training program (https://www.readvizamyl.com/jp) developed by GE Healthcare for the interpretation of ^18^F-flutemetamol images and were certified by the Japanese Society of Nuclear Medicine after passing a subsequent visual interpretation training program. The two readers were blinded to clinical information and independently interpreted the PET images according to the training program instructions. Images were scaled to 90% of the pons activity using rainbow color scaling. A negative scan has more radioactivity in the white matter than in the gray matter, creating clear gray/white matter contrast. Conversely, a positive scan has at least one of the five key regions (the posterior cingulate gyrus and precuneus, frontal cortex, lateral temporal cortex, parietal cortex, and striatum) in which gray matter radioactivity is as intense as, or exceeds, that in the adjacent white matter. Findings of visual interpretation were scored as follows: grade 0, no increase in the five key regions; grade 1, increased accumulation in one of the five key regions; and grade 2, increased accumulation in two or more of the five key regions. The two readers shared their results. If the two readers reached different conclusions, the visual rating was rerun until the readers reached consensus for each case. In a rerun, the gray matter radioactivity in five regions was more carefully compared with that in the adjacent white matter.

**Figure 1:**
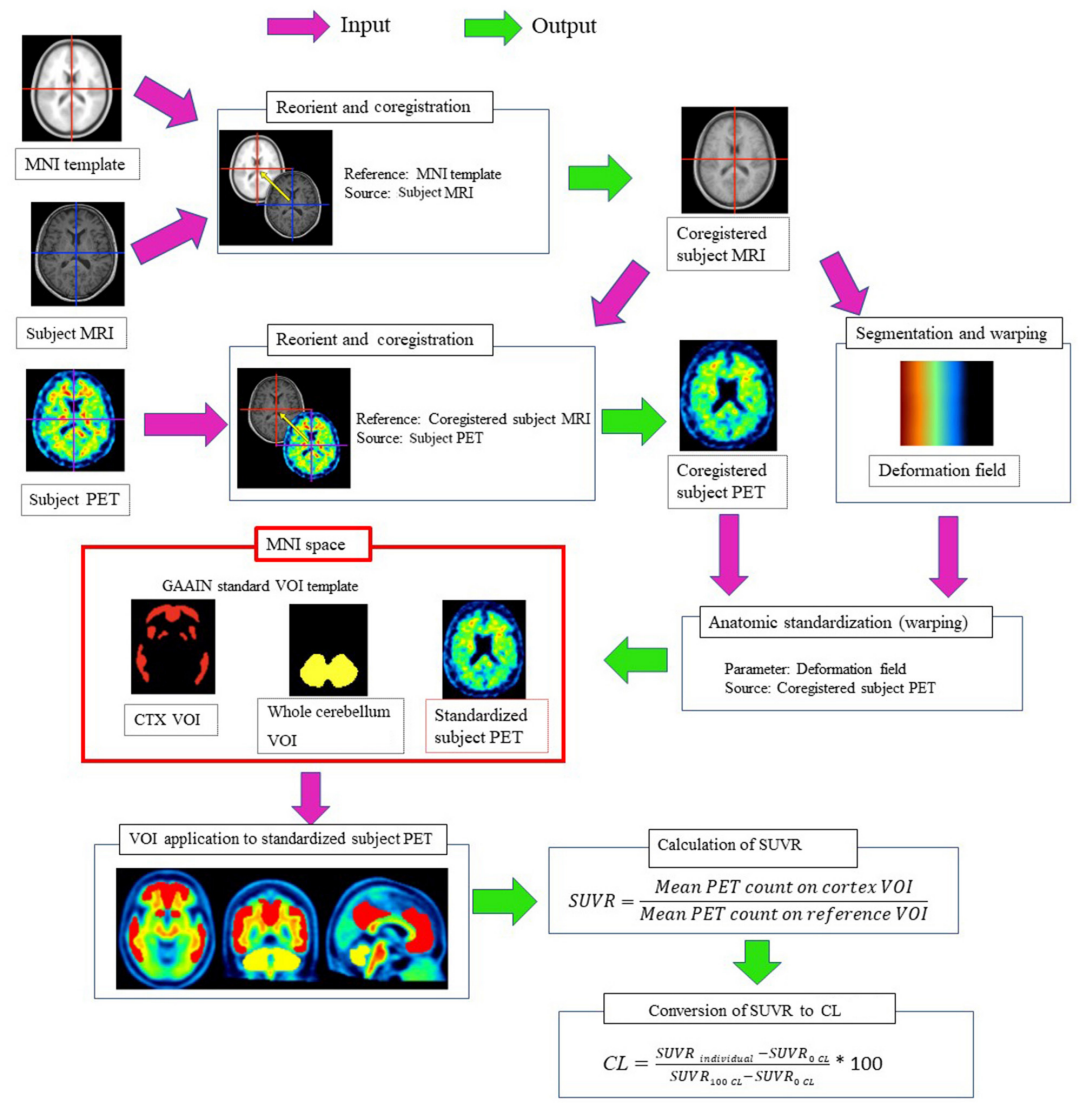
Processing pipeline for quantitative measurements of ^18^F-flutemetamol accumulation in the cerebral cortex. The subject MRI was manually oriented and coregistered to the MNI template. The subject PET was manually oriented and coregistered to the coregistered subject MRI. Then, the coregistered subject MRI was warped into MNI space using *unified segmentation* in SPM12. The parameters of the deformation field in this warping were applied to the coregistered subject PET for anatomical standardization into MNI space. The SUVR was calculated from ^18^F-flutemetamol PET counts in the cerebral cortical areas (GAAIN CTX VOI) and in the whole cerebellum (GAAIN Whole cerebellum VOI) as a reference region using CL standard volumes of interest. Then, the SUVR was converted to CL using a direct conversion equation.

The processing pipeline for quantitative analysis using the SUVR and the 100-point CL scale (10) is illustrated in Figure 1. The CL scale assigns an average value of zero in high-certainty amyloid-negative subjects and an average of 100 in typical AD patients. This pipeline was first validated using the Global Alzheimer's Association Interactive Network (GAAIN) dataset of ^11^C-PiB PET images for 34 young control individuals and 45 typical AD patients downloaded from the GAAIN website (http://www.gaain.org/centiloid-project). First, the subject MRI was manually oriented and coregistered to the Montreal Neurological Institute (MNI) template (avg152T1.nii) provided with Statistical Parametric Mapping (SPM) 12 software (https://www.fil.ion.ucl.ac.uk/spm). The subject PET was then manually oriented and coregistered to the coregistered subject MRI. Then, the coregistered subject MRI was warped into MNI space using *unified segmentation* in SPM12. The parameters of the deformation field in this warping were applied to the coregistered subject PET for anatomical standardization into MNI space. The standardized SUVR was calculated from ^18^F-flutemetamol PET counts in the global cortical target region (GAAIN CTX VOI) and in the whole cerebellum (GAAIN Whole cerebellum VOI) as a reference region using CL standard volumes of interest (http://www.gaain.org/centiloid-project). Then, the SUVR was converted to CL values using a direct conversion equation (CL = 121.42 × SUVR – 121.16), as described in a previous report (11).

### Endpoints

The primary endpoint in this study was determination of cutoffs for quantitative assessments that showed the best agreement with positive or negative results obtained via visual interpretation of ^18^F-flutemetamol PET images. The secondary endpoint was a change in diagnosis from AD to non-AD and *vice versa* between pre- and post-amyloid PET scans. Visual interpretation findings were either positive or negative. The likelihood of AD diagnosis was classified into five categories: very high, high, moderate, low, and very low.

### Statistical Analysis

Optimal cutoff values for SUVR and CL in quantitative assessment showing the best agreement with visual interpretation were determined using Youden's index (YI) and maximal accuracy calculated from receiver operating characteristic (ROC) analysis. The cut-point derived by YI optimizes a test ability to differentiate when equal weight is given to sensitivity and specificity. It is defined mathematically as: YI = sensitivity + specificity – 1 (16).

Agreement between visual and quantitative assessments of the ^18^F-flutemetamol classification, as well as the interrater agreement for visual interpretations, was assessed using Cohen's kappa. The proportions of patients showing changes in the diagnosis of AD were assessed using the McNemar test. Statistical tests were performed using JMP ver. 9.0.2 (SAS Institute). In ROC analysis, 95% confidence intervals (95% CIs) were derived using bootstrap methods (*n* = 5,000).

## Results

Adverse events were not observed after the administration of the tracers or immediately after the PET scan.

Validation of our local standard CL (SPM12) processing pipeline using the GAAIN dataset indicated an excellent correlation with published data. The slope of the linear fit was 1.0 with an intercept of 0.293, and *R*^2^ was 0.997. Thus, our implementation of the method was within the expected range defined by Klunk et al. (10): a slope between 0.98 and 1.02, an intercept between −2 and 2, and an *R*^2^ exceeding 0.98. This validation of our local pipeline allowed us to use the previously reported equation for direct conversion from ^18^F-flutemetamol SUVR to CL (11).

**Figure 2:**
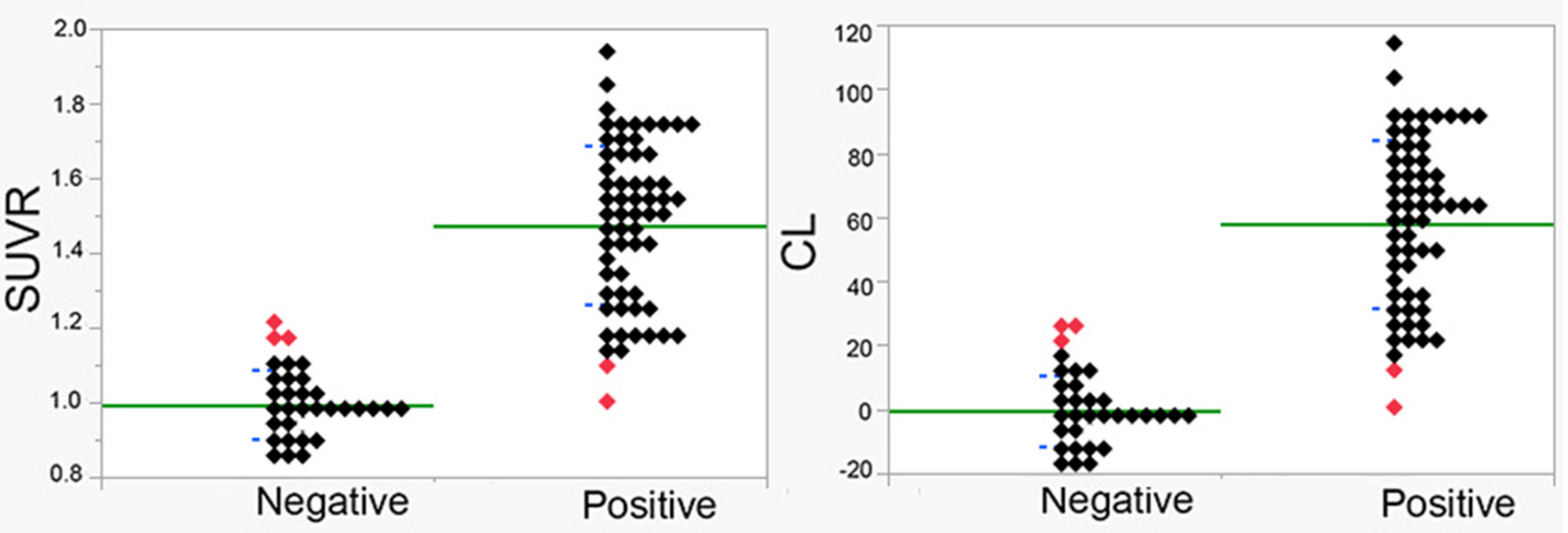
SUVR and CL values in visually amyloid-positive and amyloid-negative cases. Significant differences were observed between positive and negative cases (*p* < 0.00001). Average and standard deviation are displayed as green and blue lines, respectively. Discordant cases between quantitative measures and visual interpretation are displayed using red symbols.

MMSE score of the 93 patients was 21.2 ± 5.0 (mean ± SD) and ranged from 4 to 30. Global CDR scores were 0.5 in 52 patients, 1.0 in 33 patients, 2.0 in 7 patients, and 3.0 in 1 patient. By visual interpretation, there were 61 positive PET scans (grade 1, 10; grade 2, 51) and 32 negative PET scans (grade 0). No significant differences (Student's *t*-test or chi-square test) were found in demographic characteristics, neuropsychological evaluation, or amyloid positivity between the probable and possible AD groups (Table 2). Furthermore, there were no significant differences (Student's *t*-test) in demographic characteristics or neuropsychological evaluation between the visually amyloid-positive and -negative groups (Table 2). Amyloid positivity was observed in 30 of 52 patients (58%), 27 of 33 patients (82%), 4 of 7 patients (57%), and 0 of 1 patient (0%) with global CDR of 0.5, 1.0, 2.0, and 3.0, respectively. There was high agreement between the two readers in the visual analysis of the ^18^F-flutemetamol PET scans (85 of 93, 91.3%; Cohen's kappa = 0.82). SUVR values were 1.00 ± 0.09 and 1.47 ± 0.21 for visually negative and positive PET scans, respectively, whereas CL values were 0 ± 11 and 58 ± 26 (Figure 2). Significant differences in the SUVR and CL values were observed between visually negative and positive scans (Student's *t*-test, *p* < 0.0001). Visual disagreement between the two readers was observed in 8 cases (grade 0, 2; grade 1, 6) whose SUVR and CL values ranged from 1.10 to 1.22 and from 12 to 26, respectively. These 8 cases showed relatively mild dementia with MMSE of 23.0 ± 4.8. The interpretation results of the two readers were completely in agreement with the remaining 85 cases, which had SUVR and CL values above 1.24 and below 1.08 and above 29 and below 10, respectively.

**Table 2:** Demographic characteristics, neuropsychological evaluation, and visual assessment read of amyloid PET.

	**Probable AD (*n* = 74)**	**Possible AD (*n* = 19)**	***p*-value**
**Demographic characteristics**			
Age, years, mean ± SD	71.8 ± 11.2	72.0 ± 10.2	0.466
Sex, female/male, *n*	39/35	9/10	–
**Neuropsychological evaluation, mean ± SD**			
Global clinical dementia rating	0.9 ± 0.7	0.8 ± 0.4	0.165
Mini-mental state examination	21.2 ± 5.0	20.9 ± 5.2	0.611
Amyloid, positive/negative, *n*	48/26	13/6	0.769
	**Amyloid positive (*****n*** **= 61)**	**Amyloid negative (*****n*** **= 32)**	***p*****-value**
**Demographic characteristics**			
Age, years, mean ± SD	72.0 ± 10.8	71.4 ± 11.4	0.601
Sex, female/male, *n*	33/28	15/17	–
**Neuropsychological evaluation, mean ± SD**			
Global clinical dementia rating	0.8 ± 0.4	0.8 ± 0.6	0.524
Mini-mental state examination	21.0 ± 5.0	21.6 ± 5.1	0.278

**Figure 3:**
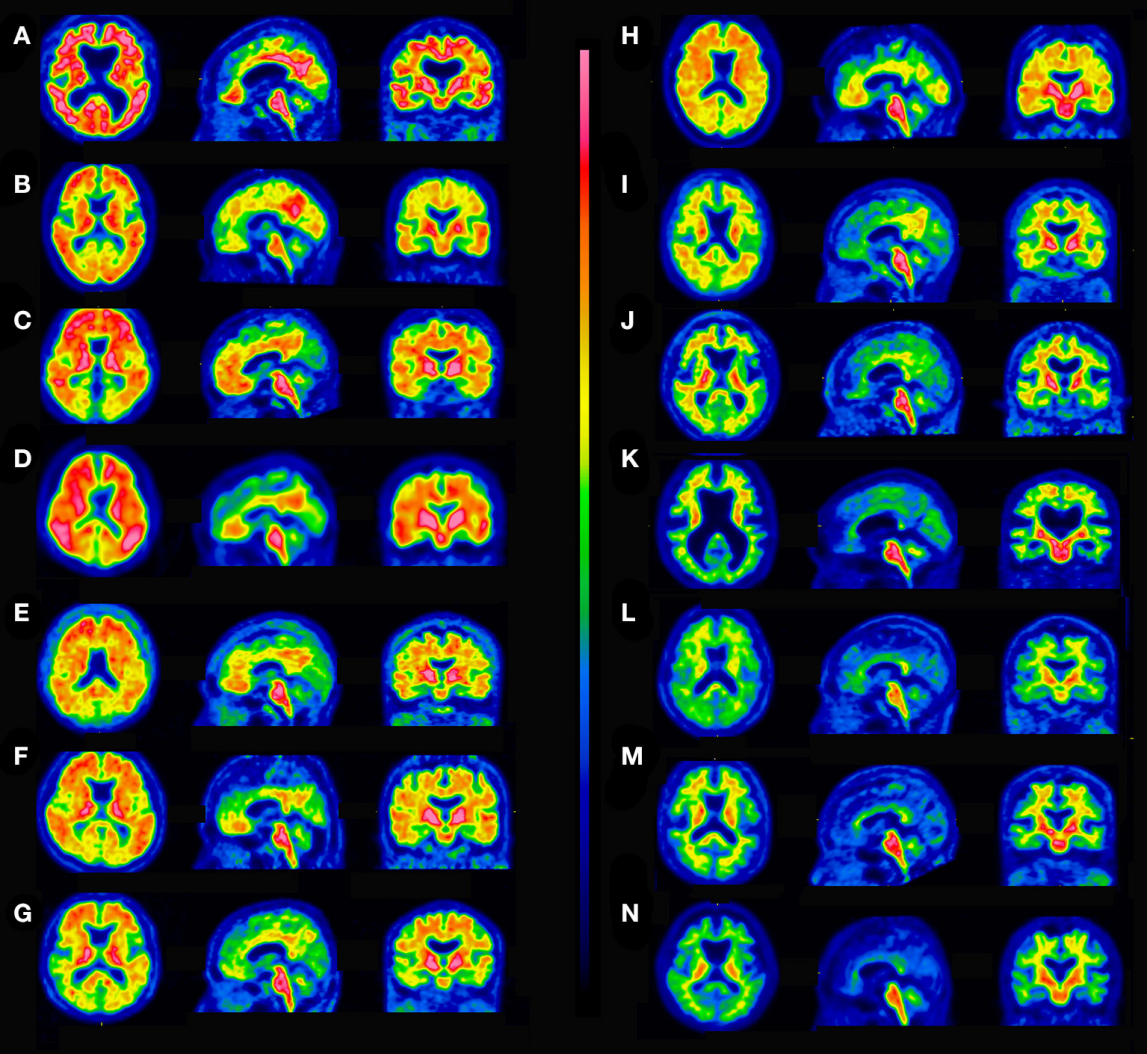
Concordant cases between quantitative assessments and visual interpretation. **(A)** Visually positive, SUVR 1.85, CL 104. **(B)** Visually positive, SUVR 1.74, CL 91. **(C)** Visually positive, SUVR 1.65, CL 80. **(D)** Visually positive, SUVR 1.57, CL 70. **(E)** Visually positive, SUVR 1.50, CL 60. **(F)** Visually positive, SUVR 1.41, CL 51. **(G)** Visually positive, SUVR 1.35, CL 42. **(H)** Visually positive, SUVR 1.24, CL 29. **(I)** Visually positive, SUVR 1.19, CL 23. **(J)** Visually positive, SUVR 1.13, CL 16. **(K)** Visually negative, SUVR 1.08, CL 12. **(L)** Visually negative, SUVR 1.04, CL 5. **(M)** Visually negative, SUVR 0.99, CL 0. **(N)** Visually negative, SUVR 0.90, CL −12.

**Figure 4:**
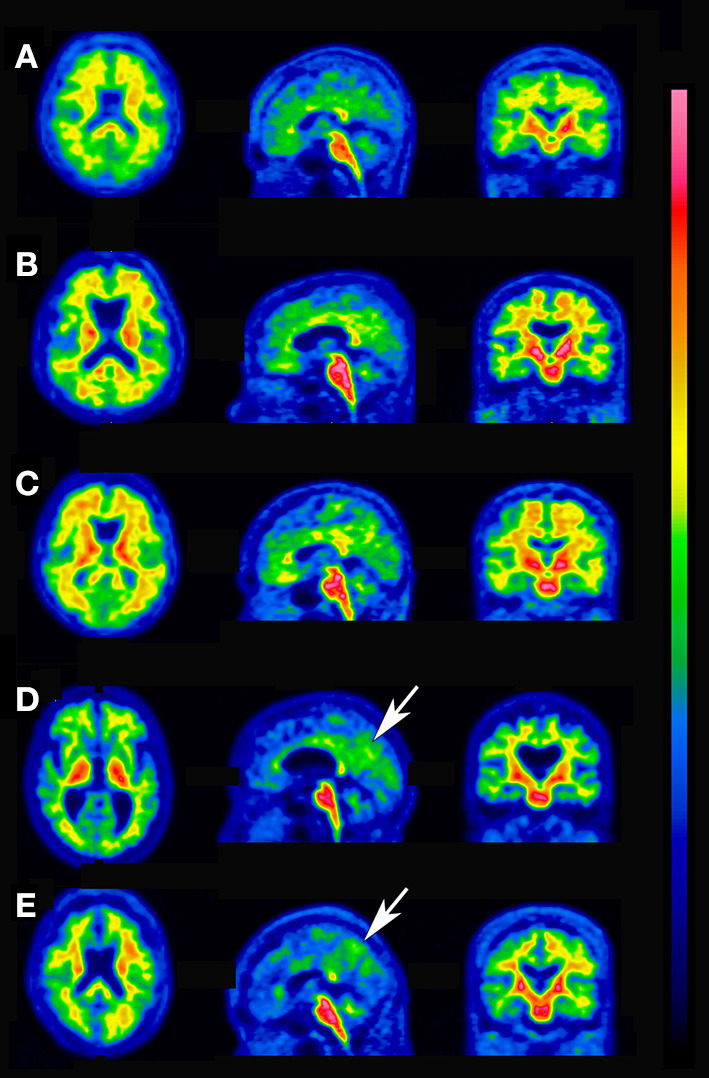
Discordant cases between quantitative assessments and visual interpretation. **(A)** Visually negative, SUVR 1.22, CL 26, diffuse elevation of cortical activity. **(B)** Visually negative, SUVR 1.19, CL 23, diffuse elevation of cortical activity. **(C)** Visually negative, SUVR 1.16, CL 19, diffuse elevation of cortical activity. **(D)** Visually positive, SUVR 1.10, CL 12, focal uptake is present in the precuneus (arrow). **(E)** Visually positive, SUVR 1.00, CL 1, focal uptake is present in the precuneus (arrow).

ROC analysis determined the best agreement of quantitative assessments and visual interpretation of ^18^F-flutemetamol PET scans to have an area under curve of 0.982 (95% CI 0.961–1.000) and a YI of 0.874 (95% CI 0.841–0.899) at an SUVR of 1.13 and a CL of 16. If visual interpretation is considered the standard of truth, quantitative assessment demonstrated 96.7% sensitivity, 90.6% specificity, and 94.6% accuracy. Using these cutoff values, there was high agreement between them (Cohen's kappa = 0.88). Cases with concordance between quantitative assessments and visual interpretation are shown in Figure 3. The SUVR and CL values of the five discordant cases between quantitative assessments and visual interpretation (Figure 4) ranged from 1.00 to 1.22 and from 1 to 26, respectively. These discordant cases were classified into two patterns. In one, diffuse elevation of cortical activity in five key areas was regarded as visually amyloid-negative (grade 0) despite relatively high SUVR or CL. In the other, mildly focal elevation of cortical activity in one area was regarded as visually amyloid-positive (grade 1) despite relatively low SUVR or CL.

**Figure 5:**
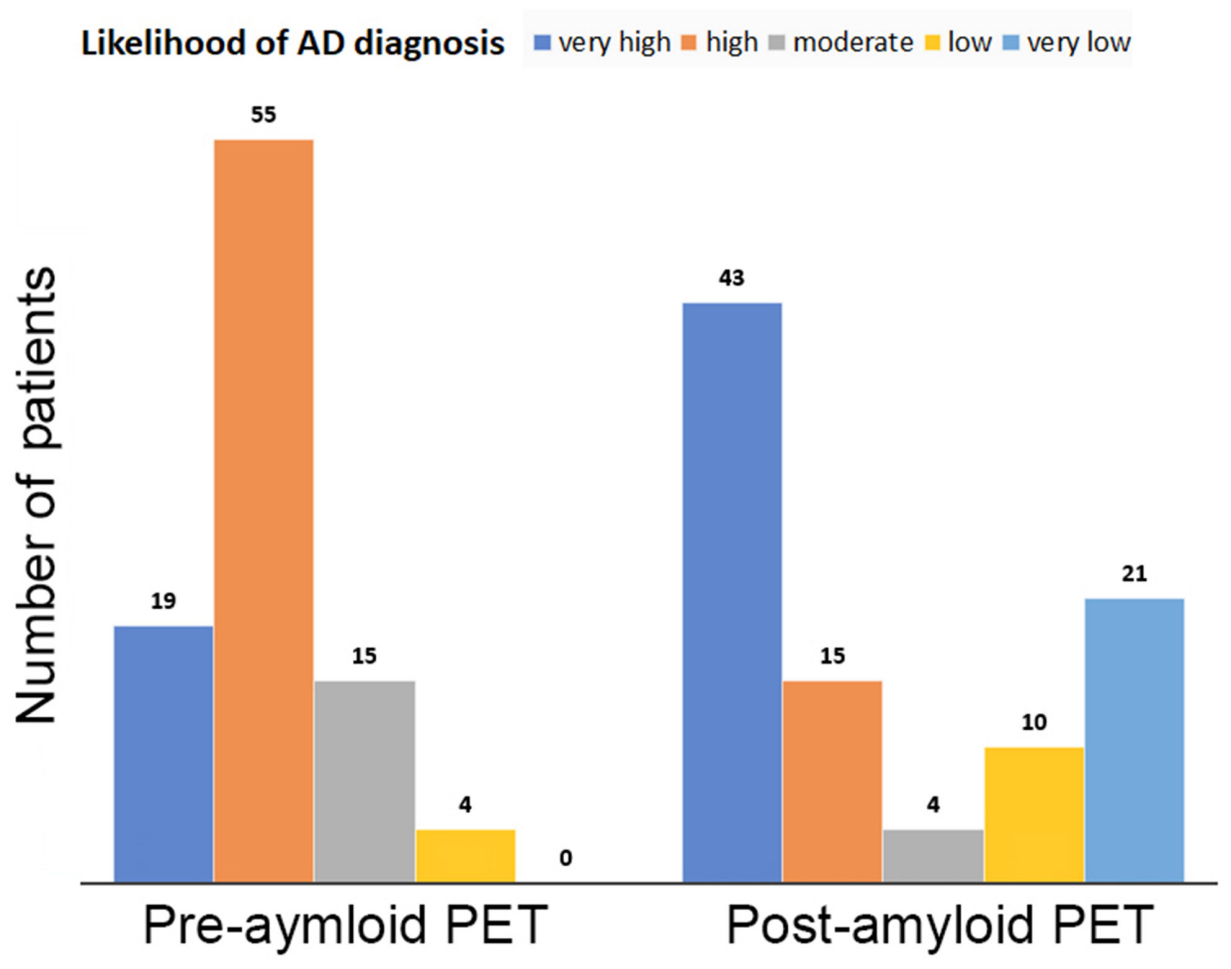
Likelihood categories of AD diagnosis before and after amyloid PET. PET scans elevated the proportions of the very high likelihood category from 20.2 to 45.7% and the very low likelihood category from 0 to 22.3%.

The amyloid PET outcome significantly altered the diagnosis of AD (χ^2^ = 51.3, *p* < 0.0001; Figure 5). PET scans elevated the proportions of the very high likelihood category from 20.4 to 46.2% and the very low likelihood category from 0 to 22.6%. In total, 73% (68 of 93) of the likelihood categories of AD diagnosis were changed after ^18^F-flutemetamol PET scanning.

## Discussion

We demonstrated a high concordance rate of 94.6% for amyloid positivity between quantitative measurement and visual interpretation categorization. This rate is lower than the 97.1% reported by Thurfjell et al. (6) for ^18^F-flutemetamol PET imaging. However, it is much higher than the 79.1% (19 of 24) in the report by Mountz et al. (7), in which four scans were rated as positive quantitatively based on the global cortical SUVR but negative qualitatively by visual classification. The authors attributed this disagreement to difficulty in differentiating a slight diffuse increase in cortical activity and high white matter uptake. This problem may explain the three visually negative scans of the five present discordant cases, even though the quantitative values exceed the cutoff. In contrast, the two other scans of the present discordant cases were visually interpreted as positive due to a focal increase in cortical activity, despite having quantitative values below the cutoff.

The present interrater agreement rate of 91.4% and kappa value of 0.82 were almost the same as those in the previous report by Hatashita et al. (8) and were better than the kappa value of 0.77 in another report by Collij et al. (9). Interrater disagreement was limited to cases in which it was difficult to judge whether or not one region showed increased accumulation, which is susceptible to interference by non-specific white matter uptake of ^18^F-flutemetamol. These instances of disagreements between readers may correspond to equivocal cases in ^11^C-PiB PET studies (3, 4). Bias in judgment criteria for visually amyloid-positive findings in at least one of the five key regions may tend to lead to deviation from the quantitative measurement in these equivocal cases. Quantitative analysis may provide more reliable results in these cases.

An SUVR of 1.35 was reported to be an ^18^F-flutemetamol-negative cutoff in the previous work (8), which is much higher than the present cutoff of 1.13. This discrepancy arose from a difference in the choice of reference areas for calculating the SUVR: cerebellar gray matter in the previous study and the whole cerebellum in the present study. The previous work also provided high SUVR values of 1.18 on average in ^18^F-flutemetamol-negative scans of young healthy controls, in contrast to the average of 1.00 in our work. This standard reference-related difference could be resolved through a CL scaling process that standardizes the quantitative amyloid imaging measures by scaling the outcome of each particular analysis method or PET ligand to a 0–100 scale. Klunk et al. (10) compared the variance and effect sizes of the difference in SUVR and CL values between the CL-100 and CL-0 groups for four standard references: the cerebellar gray matter, whole cerebellum, whole cerebellum plus brainstem, and pons. They observed that the whole cerebellum was the best standard reference, with the smallest variance and largest effect size.

Several studies have reported optimal CL cutoff values for amyloid positivity. Salvadó et al. (17) proposed an optimal cutoff value of 12 CL by comparison with the Aβ_42_ level in the cerebrospinal fluid. They also proposed that a cutoff value exceeding 29 CL denoted established AD pathology by comparison with the phosphorylated tau/Aβ_42_ ratio or total tau/Aβ_42_ ratio of the cerebrospinal fluid. On the other hand, Jack et al. (18) estimated a single cutoff value of 19 CL based on a longitudinal study. A multicenter study (19) of the relationship between standard postmortem measures of AD neuropathology and antemortem amyloid PET demonstrated that a cutoff of 12.2 CL detected CERAD moderate-to-frequent neuritic plaques, whereas a cutoff of 24.4 CL identified intermediate-to-high AD neuropathological changes. A recent similar study (20) determined that a cutoff of 20.1 CL had the highest accuracy for detecting CERAD moderate-to-frequent neuritic plaques, whereas a CL <10 was optimal for excluding neuritic plaques. That study also reported that a positive visual interpretation showed high agreement with results above 26 CL. The present comparison between CL and visual interpretation determined a cutoff of 16 CL, which is close to the cutoff for detecting CERAD moderate-to-frequent neuritic plaques. This CL cutoff value very slightly elevates sensitivity of amyloid positivity from 65.5 (61/93) to 66.6% (62/93) in the present study. On the other hand, visual interpretation for amyloid positivity by our two readers showed complete agreement with positive results above 29 CL. From these previous and present studies, visual interpretation of amyloid positivity has reliable certainty only above 26–29 CL, which may lead to reduced sensitivity of amyloid positivity.

Several studies have reported the diagnostic impact of ^18^F-flutemetamol PET in dementia. Zwan et al. (21) reported that disclosure of the amyloid PET results altered 19% of pre-PET diagnoses and increased the overall diagnostic confidence. Leuzy et al. (22) reported that ^18^F-flutemetamol PET led to a change in diagnosis in 44% of dementia patients, particularly in patients with mild cognitive impairment. Similar results were reported in a very large number of patients with mild cognitive impairment or dementia of uncertain etiology (23) in which the etiologic diagnosis changed from AD to non-AD in 25.1% of patients and from non-AD to AD in 10.5% of patients. The present study revealed that the use of PET results increased the certainty of AD and non-AD diagnoses. Amyloid PET may have added value over the standardized diagnostic workup in dementia patients with cognitive impairment and suspected AD.

This study has several limitations. First, because no postmortem data were available, the lack of a gold standard hampered our ability to relate the findings to the underlying neuropathology. Second, the sample size was not particularly large. Third, apolipoprotein E alleles that are associated with amyloid positivity were not measured in the present study. Fourth, ^18^F-flutemetamol images were interpreted by expert readers. For less experienced readers, larger interrater variability may strengthen the influence of the quantitative value on the diagnosis.

## Conclusion

We observed a high concordance rate of 94.6% for amyloid positivity between a quantitative measurement and visual interpretation for ^18^F-flutemetamol PET in patients with cognitive impairment and suspected AD. The best concordance was obtained with cutoffs of 1.13 for SUVR and 16 for CL. Quantitative analysis of amyloid PET using ^18^F-flutemetamol can objectively evaluate amyloid positivity using the present cutoffs for SUVR and CL while avoiding equivocal findings of visual interpretation. We also confirmed increased certainty of AD and non-AD diagnoses by amyloid PET.

## Data Availability Statement

The raw data supporting the conclusions of this article will be made available by the authors to any qualified researcher.

## Ethics Statement

The studies involving human participants were reviewed and approved by Certified Clinical Research Review Board at the National Center of Neurology and Psychiatry. The patients/participants provided their written informed consent to participate in this study.

## Author Contributions

HM, YS, MO, and NS generated the research idea and concept. KIt, KaI, ES, HO, MMi, SM, KeI, KO, TK, AT, HK, KH, KMa, JH, MI, TT, MMo, KIshib, and MK acquired the clinical and imaging data. HM and YS visually interpreted the PET images. TY and KMi performed the quantitative analysis. HM and TY drafted the work. HM wrote the manuscript. All authors contributed to the article and approved the submitted version.

## Conflict of Interest

HM and MMi have received research grants and speaker honoraria from Nihon Medi-Physics Co., Ltd. KaI has received a speaker honorarium from Nihon Medi-Physics Co., Ltd. KeI has received a speaker honorarium and consultant fee from Nihon Medi-Physics Co., Ltd. MK has received a scholarship donation from Nihon Medi-Physics Co., Ltd. MMi belongs to a donation course from Jusendo General Hospital. The remaining authors declare that the research was conducted in the absence of any commercial or financial relationships that could be construed as a potential conflict of interest.
